# Unveiling the domino effect: a nine-year follow-up on pentalogy of central nervous system induced by a large unruptured cerebral arteriovenous malformation: a case report and literature review

**DOI:** 10.3389/fneur.2024.1365525

**Published:** 2024-05-23

**Authors:** Yunsen He, Ye Tao, Jing Tian, Mingbin Bao, Mengjun Zhang, Qinjiang Huang, Hongliang Li, Xinxin Chang, Kun Li, Ping Liu, Lili Guo, Xiaohong Qin, Caiquan Huang, Bo Wu

**Affiliations:** ^1^Department of Neurosurgery, Sichuan Lansheng Brain Hospital and Shanghai Lansheng Brain Hospital Investment Co., Ltd., Chengdu, China; ^2^Department of Neurosurgery, Sichuan Provincial People’s Hospital, University of Electronic Science and Technology of China, Chengdu, Sichuan, China; ^3^Department of Neurosurgery, The People’s Hospital of Zhongjiang, Deyang, Sichuan, China; ^4^Sichuan Provincial Center for Mental Health, Sichuan Provincial People's Hospital, University of Electronic Science and Technology of China, Chengdu, China; ^5^Department of Neurosurgery, Wenjiang District People’s Hospital of Chengdu, Chengdu, China

**Keywords:** cerebral arteriovenous malformation, hydrocephalus, secondary Chiari malformation, syringomyelia, scoliosis, empty sella, optic disc edema, ventriculo-peritoneal shunt

## Abstract

**Background:**

The disruption of intracranial fluid dynamics due to large unruptured cerebral arteriovenous malformation (AVM) commonly triggers a domino effect within the central nervous system. This phenomenon is frequently overlooked in prior clinic and may lead to catastrophic misdiagnoses. Our team has documented the world’s first case of so-called *AVM Pentalogy* (*AVMP*) induced by a AVM.

**Clinical presentation and result:**

A 30-year-old female was first seen 9 years ago with an occasional fainting, at which time a huge unruptured AVM was discovered. Subsequently, due to progressive symptoms, she sought consultations from several prestigious neurosurgical departments in China, where all consulting neurosurgeons opted for conservation treatment due to perceived surgical risks. During the follow-up period, the patient gradually presented with hydrocephalus, empty sella, secondary Chiari malformation, syringomyelia, and scoliosis (we called as *AVMP*). When treated in our department, she already displayed numerous symptoms, including severe intracranial hypertension. Our team deduced that the hydrocephalus was the primary driver of her *AVM*P symptoms, representing the most favorable risk profile for intervention. As expected, a ventriculoperitoneal shunt successfully mitigated all symptoms of *AVMP* at 21-months post-surgical review.

**Conclusion:**

During the monitoring of unruptured AVM, it is crucial to remain vigilant for the development or progression of AVMP. When any component of AVMP is identified, thorough etiological studies and analysis of cascade reactions are imperative to avert misdiagnosis. When direct AVM intervention is not viable, strategically addressing hydrocephalus as part of the AVMP may serve as the critical therapeutic focus.

## 1 Background and importance

Imbalances in intracranial cerebrospinal fluid dynamics are prevalent disorders within the central nervous system, characterized by intricate pathways of reactions and subsequent conditions. Notably, giant unruptured arteriovenous malformation (AVM) within the cranium can cause a cascade of complications including obstructive hydrocephalus (1), empty sella syndrome (2), secondary Chiari malformation (2), syringomyelia (2), and scoliosis (2). Understanding this complete sequence may illuminate the pathological mechanisms of the disease and facilitate personalized interventions to optimize treatment outcomes at minimal costs.

Iampreechakul et al. (2) overlooked the significance of this reaction sequence, leading to severe misdiagnosis and postoperative decline. Often, conditions such as empty sella, Chiari malformation, syringomyelia, and scoliosis are mistakenly regarded as congenital issues. Previous literature has erroneously attributed the co-occurrence of these diseases within the ‘pentalogy chain’ to coincidence (2, 3). The rarity of long-term follow-ups for AVM Pentalogy (*AVMP*) and insufficient recognition of these reaction sequences have resulted in an incomplete understanding of the associated mechanisms and therapeutic approaches.

In response, we report an unprecedented case study of a supratentorial giant unruptured AVM culminating in a pentad of symptoms across a nine-year period (2014–2024). We have delved into the evolution of the supratentorial giant unruptured AVM, examining the resultant cerebrospinal fluid dynamics disturbances and their pathophysiological underpinnings. A targeted surgical intervention, devised from an understanding of this reaction sequence, yielded satisfactory outcomes.

## 2 Clinical presentation

### 2.1 Ethic approval and patient consent

We have obtained publication approval from the ethics committee of this medical institution (Sichuan Provincial People’s Hospital)^1^ as well as informed consent from the patient.

### 2.2 Basic information and chief complaint

A 30-year-old woman has been experiencing dizziness and an unsteady gait for over 8 years, coupled with headaches that began more than 2 years ago. These headaches have worsened in the past 6 months, a period marked by recurrent vomiting, blurred vision, the absence of menstruation, and numbness in her left limb.

### 2.3 Relevant past interventions with outcomes

In 2014, the patient initially sought medical attention for post-activity dizziness and subsequently received care at six tertiary neurosurgery centers across China. These centers uniformly opted for conservative management over surgical intervention, citing the elevated risk associated with surgery. Over time, the patient’s condition deteriorated, and five signs among *AVMP* (hydrocephalus, empty sella, secondary Chiari malformation, syringomyelia, and scoliosis) emerged sequentially during the course of conservative treatment. In pursuit of more specialized care, the patient was eventually referred to the neurosurgery department of our institution for the management of severe intracranial hypertension. A comprehensive account of the patient’s medical history and the progression of symptoms can be found in Table 1.

**Table 1:** The medical history of current patient.

Time	Hospital	Symptoms	Diagnosis	Management	Imaging
2014–12	The First Affiliated Hospital of Harbin Medical University	Occasional fainting	AVM	Follow-UP	Supporting materials 1
2014–12	Beijing Tiantan Hospital	Dizziness	AVM	Follow-UP	Supporting materials 1
2018–10	Shanghai Huashan Hospital	Dizziness + Headache	AVM + mild Hydrocephalus	Follow-UP	Figure 1A
2021–12	SX/Xi’an aerospace general hospital	Dizziness + Headache	AVM + mild Hydrocephalus + Partial Empty sella + moderate CMI + mild SM	Antinociception	Figures 1B,C
2022–05	Qiqihar Hospital of traditional Chinese Medicine	Dizziness + Headache + Upper extremity numbness + Menstrual disorder	AVM + severe Hydrocephalus + severe CMI + severe SM + Partial Empty sella	Antinociception+acetazolamide	Figure 1D
2022–06	Sichuan Provincial People’s Hospital (our institution)	Dizziness + Headache + Upper extremity numbness + Vomit + Blurred vision + Walking instability + Amenorrhea	AVM + severe Hydrocephalus + severe CMI + severe SM + Scoliosis + Complete Empty sella	VP shunt	Figures 1E,F
2023–12	Sichuan Provincial People’s Hospital (18 months after VP shunt)	No discomfort	AVM + Scoliosis	Follow-UP	Figures 1G–I

AVM, Arteriovenous malformation; CMI, Chiari malformation type I; SM, Syringomyelia; VP, Ventricular-peritoneal.

### 2.4 Clinical findings preoperatively

Upon admission, the patient presented with drowsiness, lethargy, vomiting, and blurred vision accompanied by papilledema. Additionally, the patient suffered from a posterior occipital headache that was exacerbated by coughing, hyperalgesia in the left limb, and a mild scoliotic deformity of the back. The preoperative assessment of the AVM yielded a Spetzler-Martin Grade of VI.

### 2.5 Medical test preoperatively

#### 2.5.1 Cranial MRI plain scan in 2014

The first original image of cranial MRI in 2014 at Affiliated Hospital of Harbin Medical University was available, but the corresponding MRI report showed only present the unruptured AVM, without hydrocephalus and other abnormalities (Supporting materials 1).

#### 2.5.2 Cranial MRI plain scan in 2018

**Figure 1:**
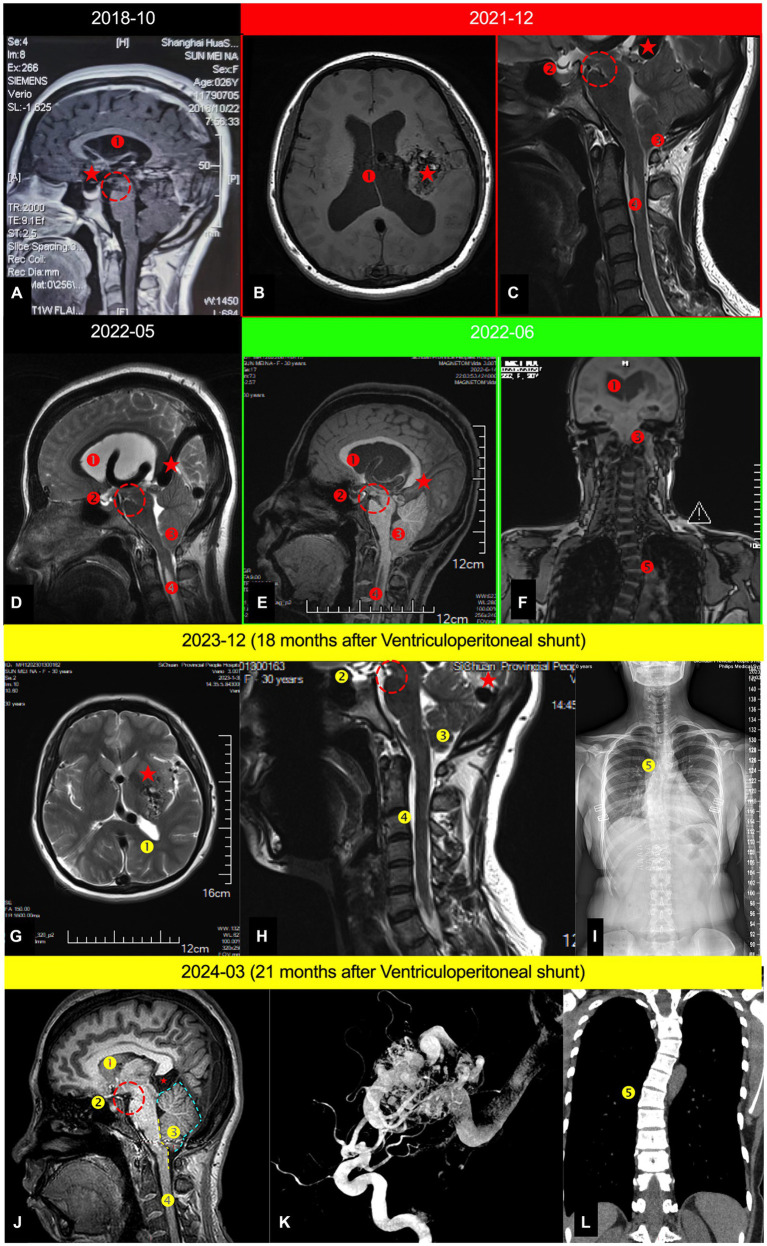
Serial cranial MRI examinations depicting the entire treatment and postoperative follow-up process of the patient; **(A)** Mid-sagittal cranial MRI in December 2018; **(B)** Axial cranial MRI in December 2021; **(C)**. Mid-sagittal cervical MRI in December 2021; **(D)** Mid-sagittal cranio-cervical MRI in May 2022; **(E)** Mid-sagittal cranio-cervical MRI in June 2022; **(F)** Coronal spinal MRI in June 2022; **(G)** Axial cranial MRI in December 2023 (18 months postoperative); **(H)** Mid-sagittal cervical MRI (18 months postoperative); **(I)** Full spine X-ray (18 months postoperative). **(J)** Mid-sagittal cervical MRI (21 months postoperative); **(K)** The digital subtraction angiography(21 months postoperative); **(L)** Coronal spinal MRI in March 2024; ⁎: AVM; ①. Hydrocephalus; ②. Empty sella syndrome; ③. Secondary CMI (Chiari malformation type 1); ④. Spinal syrinx; ⑤. Scoliosis; Yellow dashed arrow. The CSF shunt tube. Red dashed circle. Indicates the sign of midbrain sagging, referred to as the mamillopontine distance, with the specific measurement method available ④; Blue dashed line in Figure 1J represents the swollen boundary of the cerebellum; The yellow dashed line in Figure 1J delineates the boundary where the brainstem is tortuous and compressed.

In 2018, the patient visited a prestigious neurosurgery clinic in Shanghai, China, due to occasional dizziness and headaches. A follow-up cranial MRI showed no significant changes in the AVM, but revealed the emergence of mild hydrocephalus (Figure 1A). Considering the mild symptoms and the high-risk associated with surgical intervention for the AVM, the neurosurgeon recommended continued observation through follow-up visits (Table 1).

#### 2.5.3 Cranial MRI plain scan in 2020

In 2020, the patient visited a neurosurgery outpatient clinic at a hospital in Xi’an due to mild dizziness and headaches. A follow-up cranial MRI revealed increased hydrocephalus compared to previous scans, along with cerebellar tonsillar herniation (known as “secondary Chiari malformation”) (Figures 1B,C) (5, 6). Considering the risks associated with surgery for this AVM, the neurosurgeon suggested using Antinociception for oral pain relief treatment (Table 1).

#### 2.5.4 Cranial MRI plain scan in May 2022

In May 2022, the patient sought care at a neurosurgery outpatient clinic at a hospital in Xi’an due to intolerable dizziness and headaches. A follow-up cranial MRI revealed increased hydrocephalus, along with secondary Chiari malformation and a partially empty sella (Figure 1D). Considering the operative risk, the neurosurgeon recommended combining acetazolamide to reduce CSF secretion, continuously with Antinociception for oral pain relief treatment (Table 1).

#### 2.5.5 Cranial MRI plain scan in June 2022 (preoperatively)

In June 2022, the patient presented with severe dizziness, headaches, vomiting, and altered consciousness and was admitted to emergency department of our institution. A cranial MRI revealed pronounced *AVMP* throughout (Figures 1E,F). The patient was promptly transferred to the neurosurgery department for further treatment and comprehensive examinations.

#### 2.5.6 Cerebral digital subtraction angiography (preoperatively)

**Figure 2:**
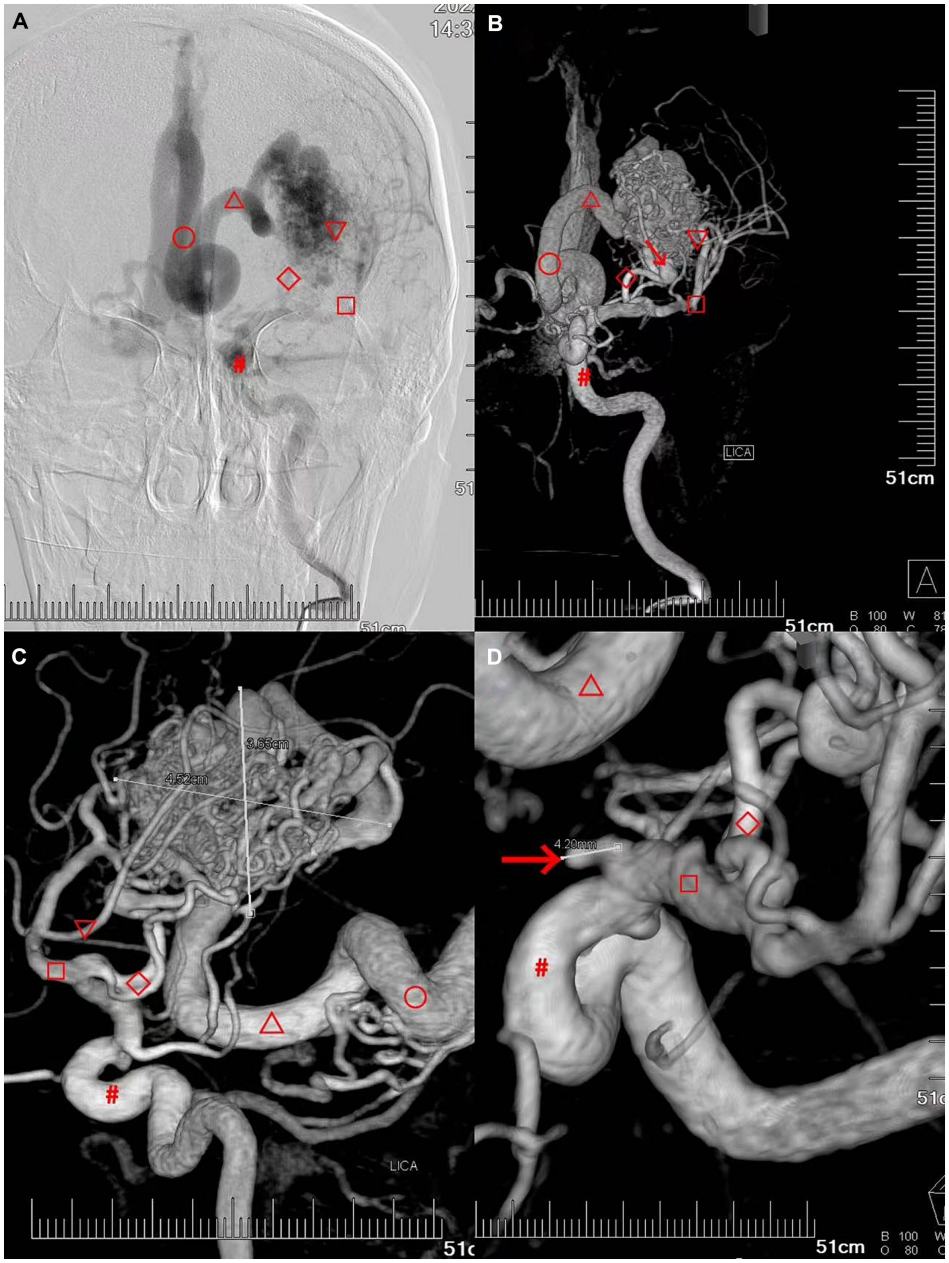
Patient’s preoperative digital subtraction angiography (DSA) of the brain. **(A–C)** Deep cerebral arteriovenous malformation in the left temporal lobe with a diameter of approximately 4.52 cm × 3.65 cm. The left deep cerebral arteriovenous malformation in the left temporal lobe was primarily supplied by the left doublestem artery (▽) the left middle cerebral artery (□) and the branch of the anterior cerebral artery (◇), draining to the straight sinus (○) via the deep vein (△) (the draining vein was dilated and blocked the foramen magnum and the three ventricles outlet). **(D)** Hemodynamic aneurysms are observed at the junction of the end of the left internal carotid artery (▽ approximately 4.2 mm) and the beginning of the M1 segment of the left middle cerebral artery (□, approximately 4.0 mm).

A whole brain angiography was performed using a Dutch Philips digital UNIQ FD20 angiography machine (DSA). The left middle cerebral artery (▽) and branches of the anterior cerebral artery (◇) from the left doublestem artery (▽) supplied the deep cerebral arteriovenous malformation in the left temporal lobe, draining to the straight sinus (○) via the deep vein (△). Two hemodynamic aneurysms were observed at the junction of the end of the left internal carotid artery (#) and the beginning of the M1 segment of the left middle cerebral artery (□) (Figure 2).

#### 2.5.7 Cranial Cine PC-MRI in June 2022 (preoperatively)

**Figure 3:**
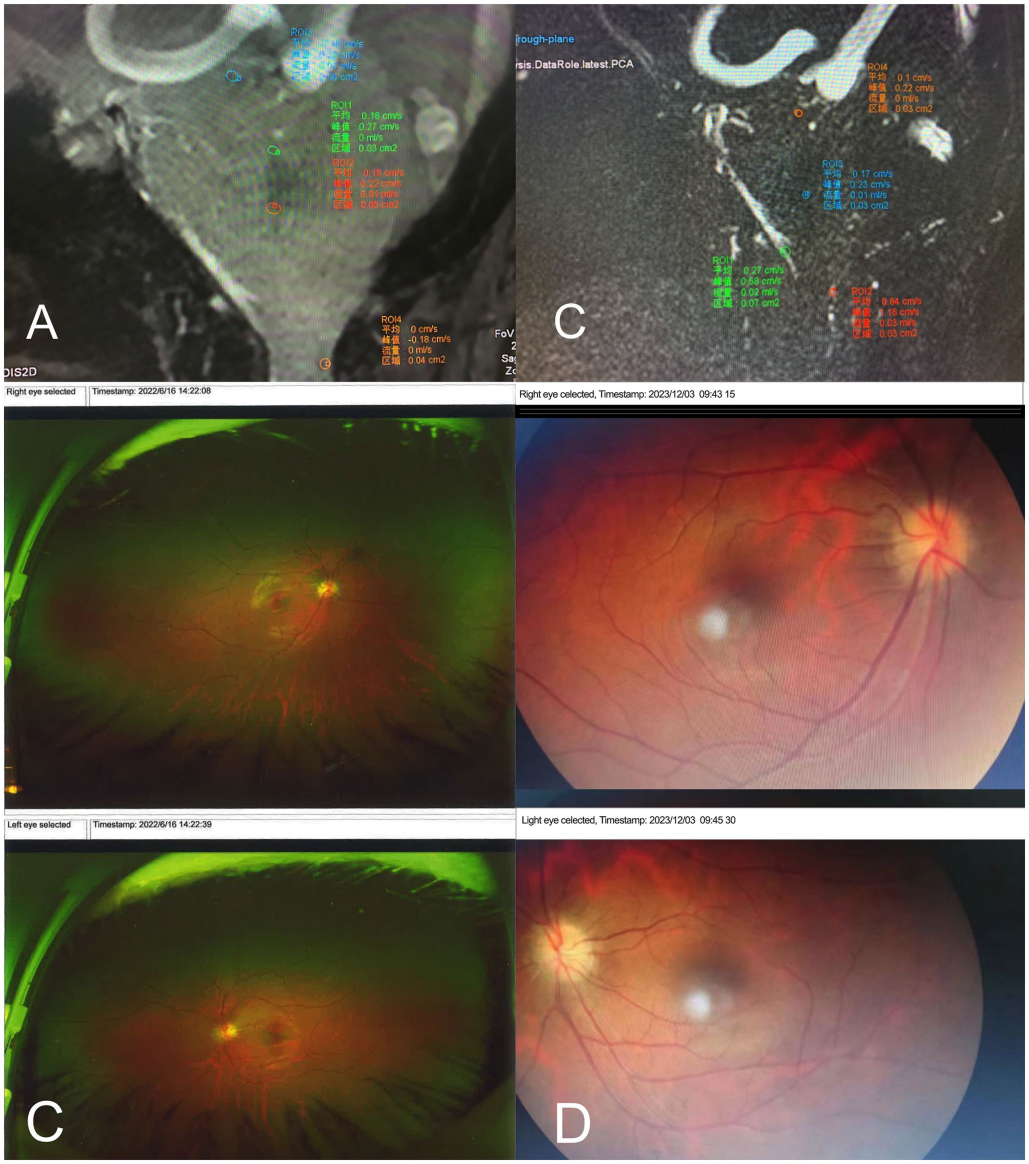
**(A,B)** The patient’s Cranial MRI with 2D-Q-FLOW sequence preoperatively measured the patient’s CVJ cerebrospinal fluid flow velocity in the mid-sagittal position. **(A)** the CSF flow velocity preoperatively in June 2022. It demonstrated an accelerated mean flow velocity in the aqueduct of sylvius (mean flow = 0.01 mL/s), the entrance to the four ventricles (mean flow = 0.00 mL/s), and the exit from the four ventricles (mean flow = 0.01 mL/s). Additionally, the flow area and total flow at the exit from the four ventricles are significantly reduced, as is the dorsal flow velocity in the craniocervical junction (mean flow = 0 mL/s). **(B)** CSF flow velocity postoperatively in December 2023. It demonstrated a slow mean flow velocity in the aqueduct of sylvius (mean flow = 0.0 mL/s), the entrance to the cisterna magna (mean flow = 0.01 mL/s), the exit from the quadrigeminal ventricles (mean flow = 0.02 mL/s), and recovery of the dorsal flow velocity in the craniocervical junction area compared to before (mean flow = 0.03 mL/s). **(C,D)** The patient’s results from ocular coherence tomography (OCT) examination. **(C)** Preoperative OCT revealed the presence of bilateral severe papilledema. **(D)**The 18 months postoperatively, and the severe papilledema was completely relieved.

A 2D-Q-FLOW sequence was performed with retrospective cardiac gating, and the patient’s CVJ cerebrospinal fluid flow rate was measured in a supine median sagittal scan. The patient’s mean CSF flow rate increased, whereas the mean flow amount decreased (Figure 3A).

#### 2.5.8 The ocular correlation tomography (OCT) in 2022 (preoperatively)

The OCT exhibited patient’s bilateral disks were congested and swollen, with flame-like hemorrhages, infarcted cotton wool-like changes in the nerve fiber layer, and heart-shaped exudates and hemorrhages in the macula. Bilateral severe progressive papilledema has been identified (Figure 3B).

#### 2.5.9 ·Laboratory testing in 2022 (preoperatively)

Pituitary hormone 6 test: the PRL = 138.14 ng/mL. All other blood tests were unremarkable.

### 2.6 Treatment

After preoperative discussion and informed consent, a regimen of intracranial pressure-lowering medication, consisting of 125 mL of mannitol administered every 8 h, was initiated to manage elevated intracranial pressure. The patient’s headache symptoms improved following this treatment. Subsequently, 3 days later, the patient underwent a ventriculoperitoneal shunt procedure for cerebrospinal fluid diversion, performed under general anesthesia in the left lateral decubitus position targeting the right lateral ventricular trigone. Intraoperatively, the intracranial pressure was monitored and found to be as high as 220 mmHg. The cerebrospinal fluid (CSF) appeared clear and colorless. An emergency CSF analysis showed no signs of infection. An adjustable Medtronic pressure ventriculoperitoneal (V-P) shunt system was implanted (Supporting materials 2), with postoperative adjustment of the shunt valve pressure based on the patient’s symptomatic presentation and dynamic cranial CT imaging.

### 2.7 Follow-up at 18 months postoperative

#### 2.7.1 ·Cranial MRI plain scan

The patient returned to our hospital 1 year later for a review: no complaints of any headache, dizziness, blurred vision, unsteady walking, vomiting, etc. MRI of the head showed: a completely alleviated obstructive hydrocephalus (Figure 1G), CMI, syringomyelia, an improvement empty sella (Figure 1H) and an unprogressive scoliosis (Figure 1I). However, the patient still exhibited significant congestion in the posterior cranial fossa, with the cerebellar tonsils notably displaced downward, although not to the extent that meets the diagnostic criteria for Chiari malformation (Figure 1J).

#### 2.7.2 ·Cranial cine PC-MRI

A 2D-Q-FLOW sequence was performed under retrospective cardiac gating, and the patient’s cranio-cervical junction (CVJ) cerebrospinal fluid flow rate was measured using a supine median sagittal scan. The results indicated that the patient’s mean CSF flow rate was slower, and the mean flow amount was higher than before (Figure 3C).

#### 2.7.3 The OCT

The follow-up examination revealed that the patient’s bilateral papilledema has completely resolved (Figure 3D).

## 3 Discussion

### 3.1 Characteristics of intracranial giant unruptured AVM

The seven previously reported cases exhibit only portions of the cascade observed in our patient (Table 2). We propose that giant unruptured AVM constitute a chronic pathology, arising from congenital vascular anomalies and persistent, progressive growth. Such unruptured AVM classified as Spetzler-Martin Grade VI are frequently encountered in clinical practice, presenting considerable challenges with limited effective management strategies. Most unruptured AVM do not cause significant long-term cerebral hemodynamic disturbances (such as epilepsy, motor-sensory impairment, and intellectual disability related to “cerebral blood steal”), due to the brain’s compensatory mechanisms in regulating cerebral blood flow De Maria et al. (7).

**Table 2:** Summary of previous cases reported by UAVM patients with chain reaction.

Author	Publication time	Gender	Age (years)	Disease course (month)	Symptoms	AVM			Hydrocephalus			CMI			Treatment	Outcome
						Location	Supply	Drain	Type	Degree	Level of obstruction	Cerebellar Tonsillar Herniation	Syringomyelia	Etiology		
Rodesch	2007	F	21	24	Headaches and loss of equilibrium	Tectal	VBs	SS	Obstructive	Moderate to severe	Aqueduct/VoG	C1	C2-C7	Hydrovenous	Four sessions of Embolized/ Stereotactic radiosurgery	Improvement
Chen	2015	F	1	3	Left upper limb weakness, vomiting, and decreased activity.	Right frontal	BACA	SSS	Communicating	Mild to moderate	Not Obstruction	Below of C1	NO	The venous hypertension of the PCF established the CVJ pressure gradient	AVM was excised	Excellent
Ogul	2017	F	3	N/A	Vomiting	Left parietal and occipital lobes	IMCA	FS (according to initial MRI)	Obstructive	Moderate to severe	Aqueduct/VoG	Below of C1	C4-C7	Dysplasia	NA	NA
Diren	2018	M	11	1	Headache, nausea and vomiting	Perimesencephalic	PAPI + CA	VoG	Obstructive	Moderate to severe	Aqueduct/VoG	C1	NO	N/A	VP shunting	Improvement
O’Shaughnessy	2006	F	19	48	Right frontal headache	Right temporal, parietal, and occipital lobes	Branches of the IMCA + PCA.	VoG	Not	Not	Not	Above of C1 (TH = 10 mm)	NO	Venous congestion and swelling of the PCF.	Embolizations/angioplasty/ excised	Improvement
Iampreechakul	2022	F	35	48	Back and right-sided sciatic pain and complete right foot drop	Right parietal and occipital lobes	Right NCA+ PCA	Superior SS /Right TS /SS and VoG	Not	Not	Not	C1	Whole-spine	Increased cerebral venous hypertension secondary to a high-flow supratentorial AVM leading to PCF venous hypertension.	PFDD/several Embolizations/stereotactic radiosurgery	Improvement
Guarrera, B.	2022	M	50s	N/A	Gait impairment, memory loss and urinary incontinence.	Dural arteriovenous fistula	Left IMA + MMA + OA + APA	The single venous drainage was through LMV and VoG	Obstructive	Not	Not	Not	Not	N/A	Embolizations/angioplasty/ excised	Improvement
Present case report	2024	F	30	48	Headache/Vomiting	Left temporal lobes	ILA + MCA + PCA	SS	Obstructive+Communicating	Moderate to severe	Aqueduct/VoG + left foramen of Monro	Above of C1 (TH = 15 mm)	C2-C6	Hydrovenous+Hydrocephalus	Ventriculoperitoneal shunt	Improvement

AVM, Arteriovenous malformation; F, Female; M, Male; NA, Not Available; MMA, Middle meningeal artery; IMCA, Ipsilateral middle cerebral artery; PCA, Posterior cerebral artery; VBs, Vertebro basilar system; BACA, Bilateral anterior cerebral arteries; PAPI, Perforating arteries of the posterior lateralartery; CA, Choroidal artery; IMA, Internal maxillary artery; OA, Occipital artery; APA, Ascending pharyngeal artery; ILA, Ipsilateral lenticulostriate arter; SS, Straight sinus; SSS, Superior sagittal sinus; FS, Falcine sinus; VoG, Vein of Galen; TS, Transverse sinus; LMV, Lateral mesencephalic vein; PCF, Posterior fossa; CVJ, Craniocervical junction; PFDD, Posterior fossa decompression duraplasty.

Nevertheless, a substantial proportion of patients may develop either the complete quintet or a subset of the secondary quintet: hydrocephalus, empty sella, acquired Chiari malformation, syringomyelia, and spinal scoliosis (Table 2), as a consequence of disruptions in cerebrospinal fluid (CSF) dynamics.

Treatment options, including direct surgical resection or vascular embolization, are associated with an elevated risk of hemorrhage and infarction during and after the procedure as noted in reference (8). For our patient, who presents with a deep unruptured AVM and a complex array of clinical manifestations, strategic removal of a critical component of the AVMP may help avert neurological deficits.

We contend that large intracranial unruptured AVM are predominantly fed by the internal carotid artery and its branches. These AVM are often accompanied by aneurysms that are hemodynamically linked and drain into the deep venous system, culminating in the straight sinus before reaching the superior sagittal sinus. This pathway may disrupt the normal absorption of CSF.

**Figure 4:**
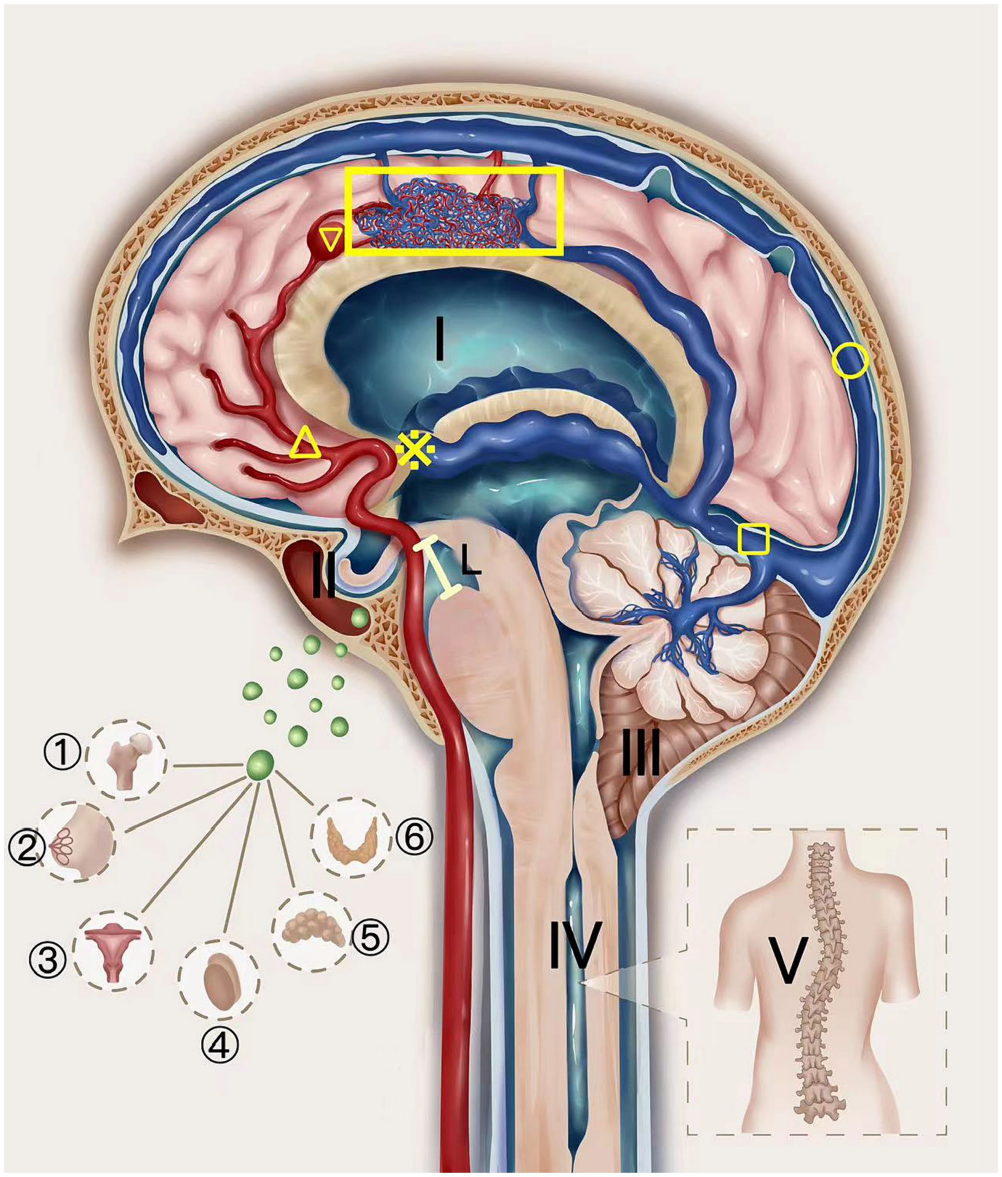
The schematic diagram of AVM Pentalogy. The UAVM depicted in the figure was supplied by the internal carotid artery and its branches (△) and was associated with an aneurysm (▽), which drained through the deep veins to the straight sinus (□) and eventually into the superior sagittal sinus (○). The thickened and tortuous drainage deep veins block the entrance of the third ventricle (※), increasing cerebrospinal fluid pressure and hydrocephalus (I), which gradually progresses to cause empty sella (II) and compresses the pituitary gland, thus affecting the hormone secretion function of the pituitary gland. This can be manifested as femoral head necrosis, nulliparity, abnormal gonadal function, adrenal disease, and thyroid disease. Increased venous pressure can also cause the midbrain to sink (Line L) (4), forcing the cerebellar tonsils to move downward to form a sub-cerebellar tonsil hernia (secondary Chiari-malformation) (III), thereby causing a syringomyelia (IV) and scoliosis (V).

Elevated venous pressure leads to the deep venous system becoming engorged and tortuous, which can obstruct the entry to the third ventricle and elevate CSF pressure, subsequently causing hydrocephalus. Intriguingly, despite these changes, the intracranial pressure may remain within normal limits initially, owing to the ventricles’ adept compliance and the brain’s intrinsic ability to self-regulate pressure at the disease’s incipient stage. As the condition advances, however, ventricular compliance diminishes, and the lack of compensatory mechanisms precipitates an increase in intracranial pressure. This escalation might result in the dilation of the third ventricle, exerting pressure on the subarachnoid space, encroaching upon the pituitary fossa, leading to an empty sella syndrome, and compressing the pituitary gland, which disrupts hormone secretion. Consequently, a spectrum of endocrine dysfunctions may manifest, including femoral necrosis, amnesia, gonadal irregularities, adrenal disorders, and thyroid conditions. The pressure can propagate to the posterior cranial fossa, potentially heightening venous pressure in this region, impeding venous return, contributing to brain tissue congestion and edema, along with midbrain descent (4). This process can compel the cerebellar tonsils to descend, resulting in tonsillar herniation (Chiari malformation), which in turn may induce syringomyelia and consequent scoliosis (Figure 4).

We posit that unruptured AVM represent a chronic pathology, stemming from congenital aberrations in cerebral vasculature that persistently enlarge throughout the patient’s lifetime. In scenarios where direct surgical intervention for unruptured AVM presents significant risks, strategic removal of a pivotal element in the pathological cascade—such as the alleviation of hydrocephalus—might forestall the evolution of neurological deficits (9).

### 3.2 Mechanism of unruptured AVM pentagram

#### 3.2.1 ·Unruptured AVM and hydrocephalus

Initially, dilation of draining veins can lead to the compression of narrow passageways within the ventricular system, such as the foramen of Monro or the aqueduct of Sylvius (1, 10–14), typically resulting in obstructive hydrocephalus. This form accounts for 94.4% of such presentations, characterized by moderate to severe hydrocephalus and representing approximately 72% of all identified mechanisms (3, 11). Secondly, chronic venous hypertension from impaired drainage can hinder the reabsorption of cerebrospinal fluid (CSF), usually leading to communicating hydrocephalus (15). This is observed in almost all cases, where the hydrocephalus is primarily mild to moderate, constituting about 28% of the mechanisms (7, 16).

In our patient’s case, both mechanisms of hydrocephalus were likely at play. The initial CT scan indicated mild hydrocephalus, presenting with symptoms of vertigo and headache, potentially due to increased venous pressure from the AVM and compromised CSF absorption. As the condition progressed, the patient exhibited symptoms indicative of severe hydrocephalus, such as vomiting and blurred vision, which may correlate with the development of non-communicating hydrocephalus. This could be attributed to the thickening of the draining vein, leading to complete obstruction of the Sylvius aqueduct or, less commonly, the foramen magnum.

#### 3.2.2 ·Primary empty sella

Dhandapani (17) posits that chronic increased intracranial pressure is a likely cause of empty sella. The proposed mechanism involves the enhanced compliance of the ventricular system due to hydrocephalus and intracranial hypertension, which allows CSF to encroach upon the pituitary fossa, resulting in vacuolation of the sella. Consequently, this may lead to hyperprolactinemia-associated symptoms such as lactation, menstrual irregularities, and amenorrhea. In the case of our patient, amenorrhea was the sole presentation in 2022, coinciding with radiological evidence of an empty sella (Figure 1H).

Pre-operative prolactin levels were elevated at 138.14 ng/mL, while post-operative levels decreased to 11.35 nmol/L. Despite minimal radiological improvement, the alleviated pressure on the pituitary gland led to a gradual restoration of its endocrine function. Reviewing prior reports, we noted instances where patients exhibited a significant empty sella, which authors either overlooked or dismissed as coincidental (2, 18). This suggests that pituitary hormone disorders in these patients might have been underestimated or entirely missed.

#### 3.2.3 ·Chiari malformation type I (CMI), syrinx, and scoliosis

CMI is primarily recognized as a congenital disorder characterized by a reduced posterior cranial fossa (PCF) size, which is attributed to mesodermal dysplasia (19–21). Yet, recent literature suggests that acquired factors may also play a role in the pathogenesis of this condition (18). Fric et al. (22) proposed that intracranial hypertension could lead to a widespread secondary form of CMI, known as Chiari syndrome. Surgical decompression and duroplasty are the recommended treatments for congenital CMI, while acquired CMI should be managed by addressing the underlying etiology. Identifying the precise cause of acquired CMI is crucial for effective management, and treatment should be etiology-specific.

Iampreechakul (2) described a case involving a 35-year-old woman afflicted with a sizable right parieto-occipital AVM. The author initially detected tonsillar herniation concurrent with syringomyelia during a routine cervical MRI, which was presumed to be a “congenital Chiari malformation (5),” leading to an Atlanto-occipital decompression. Post-surgery, however, the patient’s condition deteriorated. Subsequent brain MRI uncovered a large unruptured AVM, establishing the AVM as the initial cause; this prompted a vascular intervention, which ameliorated the patient’s symptoms.

In our case, no congenital cranial abnormalities were found on cranial MRI. The initial brain imaging from 2014 and a subsequent MRI in 2018 (Figure 1) displayed only mild hydrocephalus without any indication of tonsillar herniation or syringomyelia. However, as the hydrocephalus progressed, tonsillar herniation, syringomyelia, and even scoliosis emerged, indicative of an acquired CMI.

Moreover, previous studies have suggested that AVM can induce tonsillar herniation and syringomyelia through venous hypertension of PCF. O’Shaughnessy et al. (23) documented the inaugural case of a unruptured AVM leading directly to tonsillar herniation without accompanying hydrocephalus in 2006, with venous congestion and PCF structure swelling due to venous hypertension posited as contributing factors (23). This results in increased PCF pressure, causing tonsillar herniation, the so-called CMI, extended herniation, syringomyelia, and scoliosis, which are evidently not congenital deformities (24).

Although there have been five reported cases of AVM coexisting with CMI, the mechanism by which AVM lead to CMI remains highly contentious. Some scholars believe it is due to hydrocephalus causing midbrain sagging (4), while others attribute it to venous hypertension leading to swelling in the PCF (25). Remaining reports either arbitrarily deem it coincidental (3, 26) or overlook this diagnosis altogether (9). However, our analysis of current case suggests that both midbrain sagging and increased pressure in the PCF might contribute to the downward displacement of the cerebellar tonsils. This is supported by prior MRI findings in the patient showing simultaneous cerebellar tonsillar herniation and midbrain sagging (Figures 1A, C–E). After comprehensively addressing the hydrocephalus with a ventriculoperitoneal (V-P) shunt surgery, a significant rebound of the cerebellar tonsillar herniation was observed (Figures 1H,J), yet it remained below the craniocervical junction (CCJ; Figures 1H,J), with persistent abnormal swelling in the PCF and notable compression of the brainstem (Figures 1H,J). Therefore, we propose that for *AVMP*-associated CMI, hydrocephalus plays a dominant role, while the direct impact of AVM on CMI is secondary.

### 3.3 Treatment

The long-term progression of untreated giant unruptured AVM and associated lesions is not well-documented. Ventriculo-abdominal shunts have previously been reported as producing favorable outcomes in treating hydrocephalus without addressing inoperable and high-risk AVM (1, 27).

In managing this case involving a large, high-risk unruptured *AVMP*, we utilized a ventriculoperitoneal (V-P) shunt to alleviate intracranial pressure, effectively mitigating the classic triad of intracranial hypertension: headache, vomiting, and optic papilledema. This intervention not only interrupted but seemingly reversed the disease’s trajectory. 21 months post-surgery, follow-up MRI revealed complete resolution of the tonsillar herniation and syringomyelia, with the remaining findings being an improved empty sella and stable scoliosis.

Postoperative assessments showed a normalized prolactin level of 11.35 ng/mL, and the patient’s menstrual cycle resumed. Yet, it is plausible to consider that had a V-P shunt been implemented earlier in the hydrocephalus development, irreversible conditions such as the persistent empty sella and scoliosis might have been avoided.

The management of hydrocephalus secondary to unruptured AVM remains a topic of debate. Champeaux (28) described a case where an endoscopic third ventriculostomy (ETV) was utilized for a unruptured AVM-induced hydrocephalus that eventually led to recurring and exacerbating symptoms following an initial V-P shunt. The complications were hypothesized to relate to the considerable size of the unruptured AVM, recurrent V-P shunt malfunctions, and a suspected infection. However, we advocate for the V-P shunt over ETV in unruptured AVM-associated hydrocephalus for several reasons. Primarily, the presence of draining veins within large AVM advises against deep brain manipulation due to the risk of iatrogenic hemorrhage. Additionally, untreated AVM might progress and potentially re-occlude the ventriculostomy. Furthermore, hydrocephalus related to AVM often involves venous hypertension, which hinders CSF absorption by the central nervous system. The V-P shunt, with its ability to progressively regulate pressure via the shunt pump, offers a gradual pressure release compared to the immediate relief provided by ETV, which could pose a significant rupture risk for AVM. Although the literature hints at ETV and V-P shunts being potentially lower-risk interventions, conclusive evidence is still pending (29). In this particular case, the patient’s hydrocephalus was successfully managed with a V-P shunt, and subsequent follow-up indicated a favorable prognosis.

The treatment of patients with *AVMP* should adhere to the principle of maintaining pressure equilibrium within the central nervous system. According to the theory proposed by Lang et al. (30), alterations in the pressure/volume relationship between intracranial pressure, the volume of CSF, blood, and brain tissue can compromise neurological integrity. Therefore, in managing intracranial hypertension in *AVMP* patients, efforts should be made to maintain normal fluid dynamics and CSF resorption. Consequently, employing a V-P shunt to control hydrocephalus is effective. However, excessive drainage should be avoided, as it can lead to dangerous shifts in the midline structures, causing further neurological deterioration.

## 4 Limitation

1. Some imaging data were lost due to the prolonged duration of the disease.
2. The postoperative follow-up is relatively short, at only 18 months.
3. The patient’s AVM has not been completely treated; although she is currently in complete remission, her condition will continue to be monitored for any future progression.

## 5 Conclusion

We present a comprehensive review of the pioneering concept of AVM pentalogy through the long-term follow-up of a patient with a supratentorial giant unruptured AVM over a nine-year period. Unruptured AVM can precipitate intracranial hypertension and diverse forms of hydrocephalus, subsequently resulting in empty sella, secondary Chiari malformation, syringomyelia, and scoliosis. This review also delves into the underlying pathophysiological mechanisms. Furthermore, it serves as a valuable resource in mitigating the risks of underdiagnosis or misdiagnosis and guides clinicians toward more informed treatment decisions.

## Data availability statement

The original contributions presented in the study are included in the article/Supplementary material, further inquiries can be directed to the corresponding authors.

## Ethics statement

The studies involving humans were approved by Sichuan Provincial People’s Hospital (https://www.samsph.com). The studies were conducted in accordance with the local legislation and institutional requirements. Written informed consent for participation in this study was provided by the participants’ legal guardians/next of kin. Written informed consent was obtained from the individual(s) for the publication of any potentially identifiable images or data included in this article.

## Author contributions

YH: Writing – original draft, Writing – review & editing, Conceptualization, Data curation, Formal analysis, Funding acquisition, Investigation, Methodology, Project administration, Resources, Software, Supervision, Validation, Visualization. YT: Data curation, Formal analysis, Funding acquisition, Investigation, Resources, Supervision, Validation, Visualization, Writing – original draft, Writing – review & editing. JT: Funding acquisition, Investigation, Resources, Supervision, Validation, Writing – original draft, Writing – review & editing, Conceptualization, Methodology, Project administration. MB: Investigation, Methodology, Resources, Supervision, Validation, Writing – original draft, Data curation, Formal Analysis. MZ: Data curation, Investigation, Methodology, Writing – original draft, Project administration, Visualization. QH: Data curation, Investigation, Methodology, Project administration, Writing – original draft, Conceptualization, Formal analysis, Resources. HL: Conceptualization, Formal analysis, Investigation, Methodology, Resources, Writing – original draft, Validation. XC: Investigation, Methodology, Resources, Data curation, Funding acquisition, Project administration, Writing – review & editing. KL: Investigation, Methodology, Resources, Conceptualization, Formal analysis, Visualization, Writing – review & editing. PL: Formal analysis, Methodology, Resources, Visualization, Data curation, Project administration, Software, Writing – review & editing. LG: Data curation, Formal analysis, Project administration, Resources, Funding acquisition, Investigation, Validation, Writing – review & editing. XQ: Funding acquisition, Investigation, Conceptualization, Methodology, Writing – review & editing. CH: Funding acquisition, Investigation, Project administration, Writing – original draft, Writing – review & editing. BW: Writing – original draft, Writing – review & editing.

## Footnotes

^1^ https://www.samsph.com
