# Supplementary material for: Unveiling the domino effect: a nine-year follow-up on pentalogy of central nervous system induced by a large unruptured cerebral arteriovenous malformation: a case report and literature review
Source: Front Neurol. 2024 May 23;15:1365525. doi: 10.3389/fneur.2024.1365525 (PMC11153792; doi:10.3389/fneur.2024.1365525)
Supplement: Supplementary file 1 [file Image_1.pdf]

Patient's admission records at 2014.

哈尔滨医科大学附属第二医院

住 院 志

姓名：[redacted]

姓名：[redacted]　　科室：神经外科三病房　　病案号：1129621

出生:1992-02-03　　性别:女　　年龄:22岁

国籍:中国　　职业:　　民族:汉族

身份证号:230221199202030424　　婚姻状况:未婚

出生地：黑龙江

户口地址:黑龙江省龙江县龙江镇通达街九委52组　　与患者关系:自己　　电话:13156550020

工作单位:　　邮政编码:

供史者:[redacted]　　可靠程度:可靠　　在本院第1次住院

入院时情况一般　　入院时间:2014-12-30 13:12　　记录时间:2014年12月30日 14时

主　诉：突发晕倒5日

现病史:患者于5日前突然无诱因晕倒，数分钟后自行缓解。随即到我院行MRI：脑血管畸形。患者及家属为求进一步诊治，今来到我院，我科以“脑血管畸形”收治入院。患者在发病期间偶有头痛，可自行缓解，无肢体活动不良，无二便失禁。

既往史：　平素健康状况:一般　　传染病史:无　　手术史:无

外伤史:无　　过敏史:无　　输血史:无

既往疾病:否认高血压、糖尿病史。

个人史：　出生地：黑龙江　　到过疫区:无　　冶游史:无

嗜烟:无　　戒烟:无 约（）年　　嗜酒:无

家族史：　父健康:是　　母健康:是　　子女健康：已故（死因：）　　家族传染、遗传性病史:无

注:病史记录经陈述者确认无误并签字:刘美刚 与患者关系 本人　　签字时间：2014年12月30日.

体 格 检 查

生命体征：　体温:36.3℃　　脉搏:108次/分　　呼吸:18次/分

血压:91/66mmHg

一般状况：　发育:正常　　营养:良好　　面容:无病容

表情：自如　　体位:自主　　配合检查:合作

皮肤黏膜：　色泽:正常　　皮疹:无　　皮下出血:无

毛发分布:正常　　温度与湿度:正常　　皮肤弹性:正常

水肿:无　　肝掌:无

淋巴结：　周身浅表淋巴结肿大:无

头　部：　头颅大小:正常　　畸形:无　　眼:眼睑:正常

结膜：正常　　巩膜:黄染:无　　角膜:正常

溃疡：无　　耳:耳廓:正常　　外耳道异常分泌物:无

乳突压痛:无　　听力障碍:无　　鼻:鼻翼扇动:无

异常分泌物:无　　鼻窦压痛:无　　口腔:唇:红润

哈尔滨医科大学附属第二医院

住院志

姓名：[redacted]

科室：神经外科三病房　　　　病案号：1129621

病理反射：强阳性++　阳性+　可疑±　阴性(0)

病理反射	Hoffmann征	Rossolimo征	Babinski征	Chaddock征
左	0	0	0	0
右	0	0	0	0

不随意运动：无

跟膝胫试验：无法检查

皮肤营养：正常

其他：未查

共济运动：指鼻试验无法检查

Romberg征：正常

泌汗：正常

轮替试验：无法检查

植物神经机能：霍纳氏征：无

皮肤划痕：正常

辅助检查

血常规：　未提供

凝血系列:未提供

尿常规：未提供

心电：未提供

头CT:未提供

经颅多普勒彩色超声：未提供

头MRI平扫：有（脑血管畸形）

生化:未提供

其他:未提供

病历小结

[redacted]女，22岁岁，主因“突发晕倒5日”入院，查体：BP：91/66mmHg，意识清晰，言语确切，刺痛有反应，查体合作，双瞳孔等大同圆，对光反射灵敏，颈软，心肺腹无著变，四肢肌力V级，肌张力正常，生理反射存在，双下肢病理征（-），头MRI平扫：脑血管畸形。

确定诊断：

1 脑血管畸形

主治医生诊断签名：李瑞岩　　手签：

李瑞岩

2014-12-31 08:00

初步诊断：

1 脑血管畸形

住院医生病历签名：李庆斌　　手签：

李庆斌

2014-12-30 14:26

主治医生病历签名：李瑞岩　　手签：

李瑞岩

2014-12-31 08:00

[illegible]

2014.12.31

Patient's admission records solely diagnosed as: AVM, without a diagnosis of hydrocephalus.

哈尔滨医科大学附属第二医院

出院小结

科室：神经外科三病房

姓名：[redacted] 性别：女 年龄：22岁 住院号：1128621

入院日期：2014-12-30 13:12 出院日期：2015年01月03日 入院科别：神经外科三病房 住院：4天

门诊收治诊断：脑血管畸形

临床初步诊断：脑血管畸形

临床确定诊断：脑血管畸形

入院时情况：患者于5日前突然无诱因摔倒，数分钟后自行缓解。随即到我院行MRI：脑血管畸形。患者及家属为求进一步诊治，今来到我院，我科以"脑血管畸形"收治入院。患者在发病期间偶有头痛，可自行缓解，无肢体活动不良，无二便失禁。

治疗经过：1. 完善相关检查

2. 神经营养对症治疗

出院时情况：意识清晰，言语确切，刺痛有反应，查体合作，双瞳孔等大同圆，对光反射灵敏，颈软，心肺腹无著变，四肢肌力V级，肌张力正常，生理反射存在，双下肢病理征（-）。

治疗效果：好转

出院医嘱：1. 建议继续治疗

2. 定期复查

3. 随诊

The patient's "Discharge Summary" also exclusively diagnoses: AVM, without a diagnosis of hydrocephalus.

住院医生病历签名：李庆斌 手签：[illegible]

记录时间：[illegible]5-01-03 08:00

主治医生病历签名：李瑞岩 手签：李瑞岩

记录时间：2015-01-03 08:00

哈尔滨医科大学附属第二医院 病案室

医疗表格编号1-01

1.
