# Supplementary figures and images for: Unveiling the domino effect: a nine-year follow-up on pentalogy of central nervous system induced by a large unruptured cerebral arteriovenous malformation: a case report and literature review
Source: Front Neurol. 2024 May 23;15:1365525. doi: 10.3389/fneur.2024.1365525 (PMC11153792; doi:10.3389/fneur.2024.1365525)

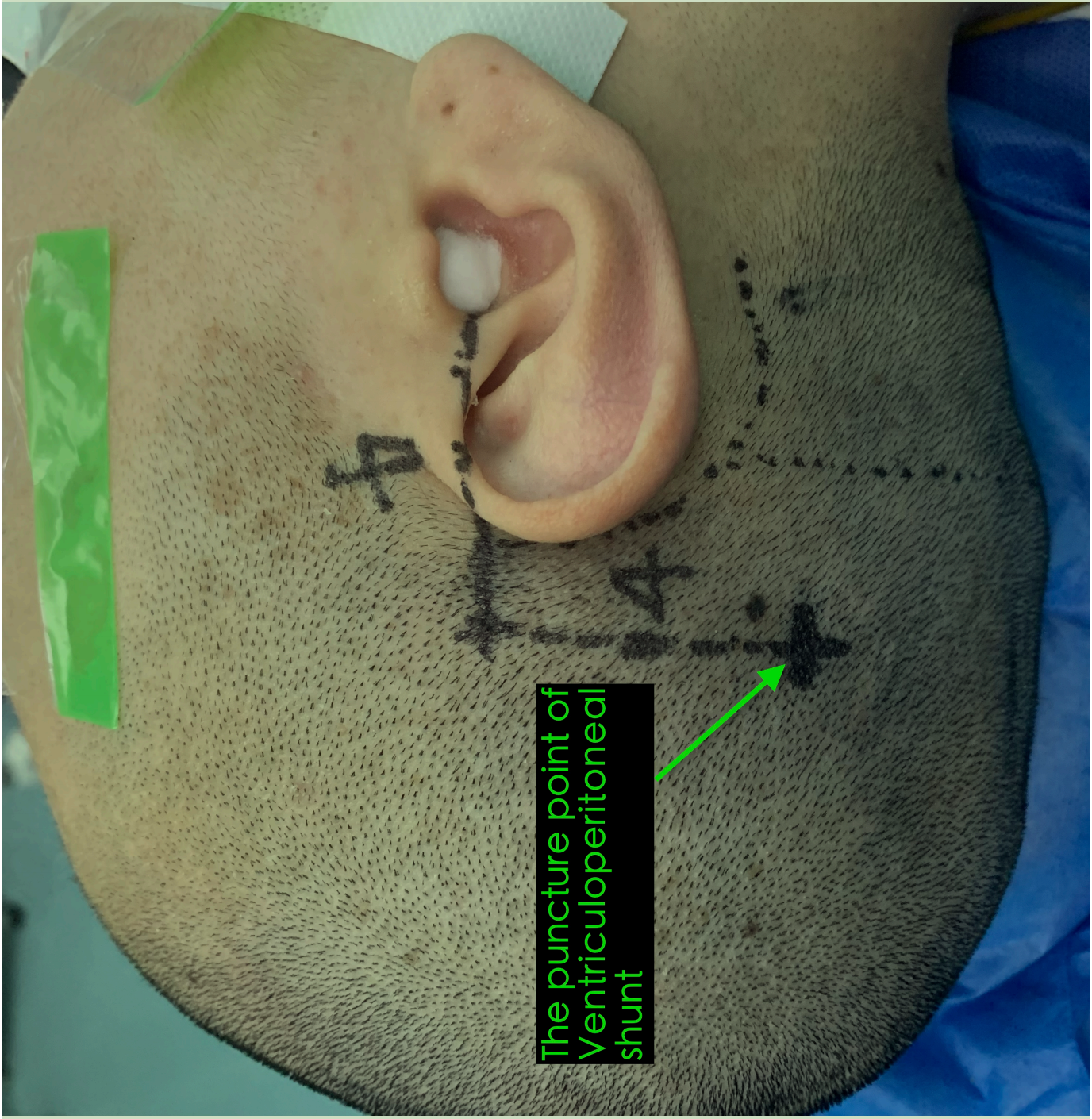

Supplement: Supplementary file 2 [file Image_2.png]
